# Socio-demographic, health-related, and individual correlates of diagnostic self-testing by lay people: Results from a representative survey in Germany

**DOI:** 10.1371/journal.pone.0188653

**Published:** 2017-11-30

**Authors:** Pinar Kuecuekbalaban, Silke Schmidt, Manfred Beutel, Kerstin Weidner, Martina de Zwaan, Elmar Braehler, Holger Muehlan

**Affiliations:** 1 Department Health and Prevention, Ernst-Moritz-Arndt-University Greifswald, Greifswald, Germany; 2 Department of Psychosomatic Medicine and Psychotherapy, University Medical Center of the Johannes Gutenberg University Mainz, Mainz, Germany; 3 Clinic and Polyclinic for Psychiatry and Psychosomatics of the University of Medical Centre Carl Gustav Carus of the Technical University of Dresden, Dresden, Germany; 4 Department of Psychosomatic and Psychotherapy of the Medical University of Hannover, Hannover, Germany; Georgia Regents University, UNITED STATES

## Abstract

**Introduction:**

A broad range of self-tests (testing for e.g. HIV, cancer, hepatitis B/C) have become available and can be conducted by lay consumers without the help of a health professional. The aims of this study were to (a) investigate the prevalence of self-testing, (b) identify the most frequently used self-tests, and (c) explore the associations between socio-demographic, health-related and individual factors with self-testing.

**Methods:**

A face-to-face plus paper-pencil cross-sectional survey was conducted. The sample consisted of 2.527 respondents who were representative of the German population in terms of the age, sex, and residence. Basic descriptive statistics and univariate logistic regression analyses were performed.

**Results:**

8.5% of the participants reported having used one or more self-tests in the past, totalling 363 self-tests, with a mean of 1.7 (min. = 1, max. = 6). The three self-tests most frequently indicated were for detecting diabetes, bowel cancer, and allergies. Self-testers were older (Nagelkerke R^2^ = .006, p < .01), had a higher BMI (Nagelkerke R^2^ = .013, p < .001) and displayed more physical and mental fatigue (Nagelkerke R^2^ = .031, p < .001) than non-testers. Self-testers also reported higher global life satisfaction values (Nagelkerke R^2^ = .008, p < .01) and a higher educational level (Nagelkerke R^2^ = .015, p < .01).

**Conclusions:**

Self-testing is fairly prevalent in Germany Given the current shortage of physicians in Germany, especially in rural areas, and recent studies on the use of self-medication, the topic of self-testing has a great practical and socio-political relevance. Future studies should investigate further predictors of self-testing (e.g. contextual, situational and individual factors) as well as the emotional consequences of testing as a layperson without the attendance of a health professional.

## Introduction

A broad range of self-tests to diagnose a particular disorder or risk factor has become available to the European public via the internet [1,2]. In fact, an internet search in 2015 on German language websites found 513 unique self-tests for 52 different conditions [3] such as tests to diagnose chronic diseases (e. g. diabetes, chronic disease of the kidneys, liver, and lungs), sexually transmitted diseases (e. g. HIV, chlamydia, gonorrhoea), infectious diseases (e. g. tuberculosis, malaria, Helicobacter pylori), allergies (e. g. house dust, cats, histamine) and cancer as well as tests for the diagnostics of 12 different psychotropic substances. These self-tests were sold by 90 companies in Germany and by other foreign companies [3]. In line with previous research, self-tests were defined in this study as: (a) tests on body materials (e.g. blood, urine, saliva), (b) initiated by the consumers themselves, and (c) conducted without the involvement of a health professional [4].

The aim of a self-test is to gather information about one′s own current health status. Thus, from the perspective of public health, health promotion and prevention research, self-testing can be thought of as a desirable and proactive self-care behaviour [2]. Additionally, self-testing is consistent with current views about consumer autonomy, self-management, and empowerment [2,5]. Further potential benefits are privacy protection, avoidance of embarrassment (e.g. when testing for HIV or chlamydia), and convenience because users do not have to visit a health professional [6]. On the other hand, criticism of self-testing has also been voiced. Some self-tests have displayed very low sensitivities, yielding false-negative test results that could lead to false reassurance, or false-positive results that could give rise to anxiety and unnecessary medical examinations [7–10]. Moreover, instruction leaflets of self-tests have been found to have limited information on reliability, follow-up behaviour, and the target group of the test [11].

The regulatory framework of using a self-test varies between different European countries and for different diseases. For example self-tests to diagnose diabetes can be brought by laypeople in pharmacies in all European countries for years. Other self-tests to diagnose life threatening diseases such as HIV can also be bought by laypeople in Great Britain and France, however, in Germany, HIV self-tests are allowed to be sold only to physicians, medical laboratories and facilities [12]. Nevertheless, interested laypeople can also buy HIV self-tests via the Internet from companies which are located outside Germany [3].

Despite their potential risks, results of a survey in the Netherlands have shown that 16% of the respondents of a Dutch internet survey confirmed the use of at least one self-test [2], while in the UK 13% of the British participants of a written survey had used a self-test at least once [13]. However, both studies were not representative surveys. In the Netherlands, 12.529 participants were recruited from an existing Dutch Internet panel called Flycatcher and data was collected with the help of a structured Internet-based questionnaire [2]. In the British survey, 5.025 eligible questionnaires were collected from 8048 adults registered with six general practices in North Birmingham and Warwickshire and Worcestershire [13].

To the best of our knowledge, neither the prevalence of self-testing, nor its associations with socio-demographic, health-related or psychological factors has been investigated in Germany so far which is the aim of the current study. In addition to sociodemographic factors, several health-related and psychological factors were included which could be of interest in association with the use of self-tests. The first factor was *self-efficacy*, which is defined as one’s belief in one’s own ability to complete tasks and reach goals. The second factor was the degree of *physical and mental fatigue*, which is the core component of burnout according to Schaufeli and Greenglass [14]. We hypothesised that participants who indicated high values in *physical and mental fatigue* (e.g. ‘How often do you feel tired?’, ‘How often are you physically exhausted?’, or ‘How often do you feel weak and susceptible to illness?’) could have used self-tests with the aim of diagnosing an actual disease. The third factor was satisfaction with one’s health (*health satisfaction*). We explored our hypothesis whether higher satisfaction with one’s own health specifically is related to a lower likelihood of using a self-test.

Thus, the aims of this study were to (a) investigate the prevalence of the use of self-tests, (b) identify the most frequently used self-tests, and (c) explore the associations between socio-demographic, health-related, and psychological factors with self-testing in a representative survey in Germany.

## Materials and methods

### Study population and procedure

The participants of the survey were recruited and the survey conducted by the market and social research company USUMA GmbH (Berlin, Germany) in Germany from February to April 2014. The sampling design resulted in a representative sample in terms of age, sex, and residence according to the federal state distribution in Germany. The selection of the sample took place in three steps. First, the Federal Republic of Germany was stratified according to districts and geospatial types so that 1.500 regional strata were developed. From these regional strata, target households were then chosen according to the random-route-method, which means that the interviewers received a street address as a starting point (also called sample point). From there every third household was noted until 18 valid private addresses per sample point had been identified according to a given route inspection plan. In the third and final step, the Kish-selection grid method was used to select the target subject within the selected households to be interviewed.

The first attempt resulted in 4.644 addresses, of which 4.607 were valid. From each household one participant aged 14 years or older was randomly chosen and contacted up to three times when they were not at home. Thus, 613 households and 55 subjects had to be excluded because they were not reached. Furthermore, 640 households and 809 subjects refused participation, 23 subjects were out of town, 28 persons were sick, and 12 interviews were not analysable. The final 2.527 valid interviews were conducted by 206 interviewers of the market and social research company USUMA GmbH, constituting approximately 12 interviews per interviewer. People who were not sufficiently familiar with the German language were excluded, thus, in a strict sense, the data is representative for the *German speaking* living population in Germany. However, only participants up to 18 years received the questions regarding the use of self-tests. Initially, the research background of the study as well as the voluntary nature and the right to revoke their participation at a later stage was explained verbally to all selected participants. In addition, an official cover letter from the contracting authorities of the research project was handed over which included a written data protection declaration that ensured strict confidentiality for all data and the accurate handling of personal identification data. All participants gave implied informed consent. Written informed consent was not sought, because in the experience of USUMA GmbH it would lead to sampling biases when trying to conduct a representative survey. Furthermore, all respondents had the chance to ask questions at any time and were aware that they could stop the survey at any time without further consequences.

### Interview guideline and questionnaire

The data regarding self-testing was assessed within a representative face-to-face *plus* paper-pencil multi-topic survey. Topics other than self-testing where investigated by other researches but will not be presented here. Face-to-face interviews were used to gather all information, except for assessing the use of self-tests. Right after the interviews, the participants were handed over a paper-pencil questionnaire, to avoid potential embarrassment in having to admit having used a self-test, e.g. in the case of sexually transmitted diseases. Thus, (a) the socio-demographic factors and (b) the health-related and psychological factors were included in the interview guideline, while (c) the use of self-tests were comprised in the paper-pencil questionnaire:

(a) *Socio-demographic factors*: Age, sex, education, employment (yes/no), income on a monthly basis and partnership status (living with or without a partner) were assessed.
(b) *Health-related and psychological factors*: First, *self-efficacy* was measured with the Short Scale for Measuring General Self-efficacy Beliefs (ASKU) [15], which consists of three items with a response scale from 1 = ‘doesn’t apply at all’ to 5 = ‘applies completely’ (*M* = 4.1, *SD* = 0.81, Cronbach′s α = .92). Second, *physical and mental fatigue* was assessed with the personal burnout scale of the Copenhagen Burnout Inventory (CBI) [16]. The response scale of its six items were from 1 = ‘never/almost never’ to 5 = ‘always’ (*M* = 2.18, *SD* = 0.77, Cronbach′s α = .92). Third, *health satisfaction* was assessed within the module ‘General Life Satisfaction’ of the ‘Questions on Life Satisfaction Modules’ (FLZM) [17], on a scale from 1 = ‘not important/unsatisfied’ to 5 = ‘extremely important/very satisfied’ (*M* = 29.24, *SD* = 6.07, Cronbach′s α = .86). Additionally, the variables weight and height were used to calculate the BMI of the participants.
(c) *Use of self-tests*. To be able to compare the use of self-tests in Germany with that in the Netherlands and Great Britain, a questionnaire was developed based on the Dutch [2] and British [13] surveys. Thus, self-testing was defined as: (a) taking a body sample independently, (b) used to diagnose a disease or a risk of a disease, (c) conducted on one’s own initiative (not recommended by a health professional), and (d) without the attendance of a health professional. Hence, blood pressure monitoring was excluded from this study. Pregnancy tests were also excluded because they do not diagnose a disease. Respondents were asked to indicate whether they (a) had not known of, (b) had known of, but never considered using, (c) had already considered using, but not done so yet, or (d) had already conducted a self-test. Participants then had to indicate from a list of self-tests for 27 different conditions which tests they had used, with extra space provided to add any tests not listed. Further, in-depth information was collected regarding the frequency of the use of the self-tests, the manner of analysis of the body samples, as well as the place of purchase of the self-test and where it was performed. The intention to use a self-test in the future was also assessed on a scale from 1 = ‘definitely not’ to 5 = ‘definitely yes’. Respondents who intended to use a self-test ‘maybe’ to ‘definitely’ in the future were requested to specify which self-test they may use.

### Statistical analyses

Basic descriptive statistics were used to describe the respondents’ socio-demographic characteristics, past and intended use of self-tests, type and frequency of self-test use, where it was purchased and conducted, and how the test results were received. *Univariate* logistic regression analyses were then calculated for *each* potential predictor *individually* and the criterion self-testing (non-self-testers = 0, self-testers = 1). Analyses were performed with IBM SPSS Statistics [18]. An alpha of .05 was applied to indicate statistical significance.

## Results

### Respondents

The total sample consisted of 2.527 respondents representative of the German population in terms of the age, sex, and residence (see Table 1). The participants’ mean age was 49.4 years (*SD* = 17.8), with age ranging from 14 to 95. More than half of the participants were female (53.4%). The majority of the participants had no or the lowest formal qualification (38.4%), while 36.8% had a higher secondary education qualification, and 12.0% had a university degree. The majority of the participants stated to have an income of up to €2.000 a month (46.9%), while 14.4% of the participants had an income of up to €3.500 and 4.3% earned more than €3.500 a month. More than half of the sample was living together with a partner (58.1%). In Table 1 the socio-demographic characteristics of the total sample as wells as according to self-testers versus non-self-testers group are displayed.

**Table 1:** Socio-demographic characteristics of the total sample, and according to self-testers versus non-self-testers group.

	Total		nST^a^		ST^a^	
	N	%	n	%	n	%
	2527	100	2311	91,45%	216	85,48%
**Sex**						
Male	1177	46.6%	1079	46.7%	98	45.4%
Female	1350	53.4%	1232	53.3%	118	54.6%
**Age**						
<= 27	351	13.9%	326	14.1%	25	11.6%
28–41	511	20.2%	483	20.9%	28	13.0%
>= 42	1665	65.9%	1502	65.0%	163	75.5%
**Education**						
No qualification/primary school (4 years)	82	3.2%	76	3.3%	6	2.8%
Lowest formal qualification (8/9 years)	889	35.2%	825	35.7%	64	29.6%
Intermediary secondary qualification (10 years)	929	36.8%	857	37.1%	72	33.3%
Higher secondary education qualification (11/12/13 years)	303	12.0%	272	11.8%	31	14.4%
University degree	255	10.1%	216	9.3%	39	18.1%
Other	69	2.7%	65	2.8%	4	1.9%
**Income**^**b**^						
no personal income	177	7.0%	169	7.3%	8	3.7%
up to €1.000	690	27.3%	634	27.4%	56	25.9%
up to €2.000	1185	46.9%	1087	47.0%	98	45.4%
up to €3.000	365	14.4%	327	14.1%	38	17.6%
up to €5.000	53	2.1%	46	2.0%	7	3.2%
more than €5.000	6	0.2%	5	0.2%	1	0.5%
no response	51	2.0%	43	1.9%	8	3.7%
**Partnership**						
Living with a partner	1442	58.1%	1310	57.7%	132	62.3%
Not living with a partner	1041	41.9%	961	42.3%	80	37.7%

Note a: nST = non-self-tester, ST = self-tester

Note b: income on a monthly basis

### Prevalence of self-testing

The majority of the respondents indicated to never before have heard of self-tests (n = 1.206, 47.7%), or to have heard of them, but never considered using one (n = 852, 33.7%). Only 6.1% of the participants had already considered using a self-test, but had never done so (n = 153). The remaining 12.5% of the participants (n = 316) stated to have conducted a self-test, but 100 of these said that they had only used a pregnancy or paternity test. Hence, 8.5% of the participants (n_ST_ = 216) had used it to diagnose a disease. Altogether, the 216 self-testers had used 363 tests, with a mean number of 1.7 tests per respondent (min. = 1, max. = 6) and 9.3% of them had used self-tests to diagnose four or more different diseases. The ten most frequently indicated self-tests were those for detecting diabetes, bowel cancer, allergies, urinary tract infection, HIV infection, disorder of the thyroid, cholesterol, disorder of the kidney, prostate cancer, and blood coagulation (Table 2).

**Table 2:** Reported use of self-tests for 33 diseases as a total and according to true self-test.

	Total self-tests		true self-tests^a^			Missing
	number	percentage of the total self-tests (n = 363)	number	percentage of the total self-tests (n = 363)	percentage of the total sample (N = 2527)	number
	**363**	**100.0%**	**159**	**43.80**	**6.29%**	**74**
Diabetes	95	26.2%	68	18.73%	2.69**%**	12
Bowel cancer	45	12.4%	19	5.23%	0.75**%**	12
Allergies	38	10.5%	12	3.31%	0.47**%**	6
Urinary tract infection	22	6.1%	12	3.31%	0.47**%**	3
Disorder of the der thyroid	18	5.0%	0	0.00%	0.00**%**	11
HIV infection	18	5.0%	2	0.55%	0.08**%**	2
Cholesterol	17	4.7%	3	0.83%	0.12**%**	8
Disorder of the kidney	13	3.6%	6	1.65%	0.24**%**	3
Prostate cancer	13	3.6%	0	0.00%	0.00**%**	5
Blood coagulation	12	3.3%	5	1.38%	0.20**%**	1
Lactose intolerance	10	2.8%	5	1.38%	0.20**%**	1
Ovulation	8	2.2%	7	1.93%	0.28**%**	1
Fertility of the man	7	1.9%	3	0.83%	0.12**%**	2
Gluten intolerance	6	1.7%	5	1.38%	0.20**%**	0
Hepatitis B or C	5	1.4%	1	0.28%	0.04**%**	0
Vaginal (yeast) infection	5	1.4%	2	0.55%	0.08**%**	1
Anaemia	3	0.8%	0	0.00%	0.00**%**	2
Loss of amniotic fluid	3	0.8%	1	0.28%	0.04**%**	0
Blood levels in general	3	0.8%	2	0.55%	0.08**%**	0
Genetic disorder(s)	2	0.6%	0	0.00%	0.00**%**	2
Helicobacter pylori	2	0.6%	0	0.00%	0.00**%**	0
Influenza	1	0.3%	0	0.00%	0.00**%**	1
Glandular fever	1	0.3%	0	0.00%	0.00**%**	1
Chlamydia	1	0.3%	0	0.00%	0.00**%**	0
Fertility of the woman	1	0.3%	1	0.28%	0.04**%**	0
General test kit	1	0.3%	1	0.28%	0.04**%**	0
Skin cancer	1	0.3%	0	0.00%	0.00**%**	0
Drug test	1	0.3%	0	0.00%	0.00**%**	0
Amalgam test	1	0.3%	1	0.28%	0.04**%**	0
Faecal specimen	1	0.3%	1	0.28%	0.04**%**	0
Blood test (e.g. alcohol disorder)	1	0.3%	1	0.28%	0.04**%**	0
Norovirus	1	0.3%	0	0.00%	0.00**%**	0
Gout	1	0.3%	1	0.28%	0.04**%**	0
Not specified	1	0.3%	0	0.00%	0.00**%**	1

Note a. A true self-test situation was defined as a situation in which the consumer is responsible for all steps of the self-test procedure (conducting, interpreting, and reacting in terms of follow-up behavior).

Of the 363 conducted self-tests, 38.4% were obtained from a general practitioner, medical specialist, or in a hospital, and 30.6% were purchased in a pharmacy. About 3% were given by colleagues, friends, or relatives. Only about 1.7% were ordered on the internet. The remaining tests were acquired by various institutions such as blood donation, alternative practitioner, health insurance, or drugstore. Information on the acquisition of 76 self-tests (21.4%) was missing. The great majority of self-tests were conducted at home (43.3%), at friends’ and relatives’ or at public places, constituting 44.1% true self-test situations in which the consumer is responsible for all steps of the self-test procedure (conducting, interpreting, and reacting in terms of follow-up behaviour, see Table 2). However, the other majority (34.4%) indicated to have conducted the self-test at the office of a general practitioner, medical specialist, alternative practitioner, or in a hospital, pharmacy, local public health office, or blood donation. These users thus may have received help with the test by a health professional when needed. The majority of self-tests immediately displayed a test result (50.1%), while for 32.8% a body sample had to be sent to a laboratory by post, and the test result was received later.

Of the 2311 participants who had never before used a self-test, about 2% (n = 45) stated to definitely or probably use a self-test in the future, and about 5% (n = 115) indicated they would perhaps do so. These 160 participants stated to be interested in all together 270 self-tests. The ten most frequently considered self-tests were those for diabetes (30.0%), bowel cancer (13.0%), allergies (9.3%), prostate cancer (6.3%), urinary tract infection (4.8%), cancer (unspecified/in general, 4.4%), general urine test (3.7%), cholesterol (3.0%), disorder of the kidney (3.0%), and hereditary diseases (3.0%).

### Correlates of self-test use in general

To investigate the relationship between self-testing and various socio-demographic, health-related, and psychological variables, *univariate* logistic regression analyses were calculated for *each predictor individually* and the criterion self-testing (see Table 3). The results show that there was a significant association between the use of a self-test with the socio-demographic factors *age* and *education*. The probability of having conducted a self-test slightly increased with age. Participants with the lowest formal qualification (8/9 years) or intermediary secondary qualification (10 years) were less likely to have ever conducted a self-test, compared to participants with a university degree. Whether the respondents were living together with a partner or not was not associated with self-test usage. Participants with a higher *BMI* were more likely to be self-testers. Furthermore, there was a significant positive association between self-testing and the subjective health-related predictor *physical and mental fatigue* showing that an increase in physical and mental fatigue was associated with a higher likelihood of using a self-test. Moreover, an increase in (a) *health satisfaction* and (b) *global life satisfaction* were significantly related with a decreased likelihood of using a self-test. Overall, the predictors *physical and mental fatigue*, *health satisfaction*, and *educational level* provided the greatest error reductions (3.1%, 1.6% and 1.5%, respectively).

**Table 3:** Univariate logistic regression analysis for the criterion “self-testing” and each single predictor^b^.

	Missing	OR^a^		95%	CI^a^	Nagelkerke R^2^	p-value
**Socio-demographic factors**							
Sex^(Ref female)^	0	.948		.717	1.255	.000	
Age	0	1.011	**	1.003	1.019	.006	**
Education^(Ref University)^	69					.015	**
Up to 4 years		.437		.178	1.074		
Up to 9 years		.430	***	.281	.657		
Up to 10 years		.465	***	.307	.706		
Up to 13 years		.631		.381	1.045		
Income^(Ref more than €1250)^	69					.004	
no personal income		.485		.232	1.012		
up to €1 250		.948		.708	1.268		
Employment^(Ref yes)^	10	.504		.233	1.091	.003	
Living together with a partner^(Ref not living together)^	44	1.210		.906	1.617	.002	
**Health-related and psychological factors**							
BMI	43	1.056	***	1.028	1.085	.013	***
Physical and mental fatigue	32	1.690	***	1.422	2.008	.031	***
Health satisfaction	5	.754	***	.663	.858	.016	***
Life satisfaction	10	.966	**	.944	.988	.008	**
Self-efficacy	21	.935		.789	1.107	.001	

Note a: Odds ratio (OR) and confidence interval (CI)

Note b: Total N = 2.527, 0 = non self-tester (n_nST_ = 2.311), 1 = self-tester (n_ST_ = 216)

Note: * p < .05, ** p < .01, *** p < .001

## Discussion

### Main findings and comparison with previous research

To investigate the prevalence as well as socio-demographic, health-related, and psychological correlates of the use of self-tests, a face-to-face plus paper-pencil survey of 2.527 representatives of the German participants was conducted. The results show that the use of self-tests in Germany was lower (8.5%) than in the Netherlands (16%) [2] and the UK (13%) [13]. However, the ten most frequently used self-tests were the same for all three countries, although the ranking varied a bit. In all three countries, self-tests to diagnose diabetes were used most frequently, while the ranking of the other most frequently used self-tests varied according to country. In second and third place tests to diagnose urine infection and cholesterol followed in the UK, while Dutch participants were self-testing most frequently for cholesterol and allergies and German self-testers used tests to diagnose bowel cancer and allergies. Reasons for the lower prevalence in Germany could be methodical aspects such as different sampling procedures or inclusion criteria. For example, the sample in this study is representative of the German population in terms of age, sex, and residence, whereas neither the Dutch nor the British sample was representative. Although, in the Dutch sample Flycatcher was used which offered the opportunity to draw a sample from the entire panel, which was representative of the Dutch population, non-respondents of the Dutch Internet survey were found to be younger and less educated than respondents [2]. Furthermore, the survey in the Netherlands was an internet survey, which could have led to an increased prevalence of self-testers compared to the general population—which was also suggested by the researchers themselves [2]. Although, the study population of the British survey was also not representative, it was still similar regarding the age and sex distribution to the population of England and Wales in 2006. However, the lowest response rates were from the youngest age groups, although, younger women had considerably higher response rates than younger men [13]. Additionally, the British survey did not include the criterion that self-tests are ‘initiated by the consumers themselves’. This could have led to an increased prevalence of the British self-testers who might have conducted a test because of a recommendation by a health professional. Other reasons for the different prevalence rates in the three countries could be administrative issues such as different accessibility, law regulations, and public campaigns. For example, in the Netherlands prevention campaigns were conducted (e.g. by the Kidney Association or the Municipal Health), which recommended the use of self-tests to diagnose kidney diseases and chlamydia, and sent those self-tests free of charge to interested laypeople [19]. Possibly this is the reason why more participants of the current study had not heard of self-tests before (47.7%), compared to the Dutch participants (36.6%).

Differences regarding the intention to use a self-test in the future were even greater. While in Germany 2% of the participants who had never used a self-test before stated to ‘definitely or probably’ use a self-test in the future, about 5% indicated they would ‘perhaps’ do so, in the Netherlands these were 17% and 54%, respectively.

With respect to the socio-demographic correlates, the results of the current survey were in line with the results of Ronda et al. [2] for the Netherlands. In both countries, self-testers were more likely to be older and have a higher BMI than non-self-testers, which could be explained by the fact that self-tests to diagnose diabetes or bowel cancer were used most frequently and are most relevant with increasing age. In contrast, self-tests to diagnose e.g. HIV, fertility, vaginal infections, or chlamydia might be more interesting for younger people, but were barely used. Additionally, all German self-testers, but only *female* Dutch self-testers were more likely to have a higher than a lower educational level.

There was also no significant association between *self-efficacy*, measured with the ASKU, and the use of a self-test. This is in line with two previous studies, which found no association between (1) *general* self-efficacy, measured with the General Self-Efficacy scale [20], and self-test use in a factorial survey with 208 participants [21], and between (2) *general* self-efficacy, measured with the ASKU and self-test use, in a cross-sectional internet survey of 505 actual self-testers and 512 non-testers in Germany [22]. In contrast, a positive association between *specific self-test-related* self-efficacy was found both in a cross-sectional internet survey of 513 Dutch self-testers and 600 non-testers [4], as well as in the abovementioned cross-sectional internet survey in Germany. This suggests that specific self-test-related self-efficacy, rather than general self-efficacy, is an important predictor of using a self-test.

This study is the first investigation of the relationship between self-rated physical and mental fatigue with self-testing. Self-testers indicated higher scores in physical and mental fatigue than non-self-testers, this suggests that participants who for example felt more often tired, physically exhausted, weak or susceptible to illness were more likely to use a self-test to diagnose a disease. Furthermore, as expected, higher satisfaction with one’s own health was related to a lower likelihood of using a self-test. However, contrary to Kim et al. [23], who found in a prospective and nationally representative panel study of U.S. adults over the age of 50 (N = 7.168) that higher life satisfaction was associated with higher use of several preventive health care services such as a higher likelihood that people would obtain a cholesterol test, in this survey a higher life satisfaction was negatively associated with the use of self-tests.

### Strengths and limitations

The strength of this study is that it is the first *representative* survey in Germany which investigated the prevalence, socio-demographic, health-related, and psychological correlates of the use of self-tests. Therefore, our results have a high external validity. Another strength is that the use of self-tests was assessed by means of a paper-pencil questionnaire, to ensure that participants would not feel uncomfortable in having to state that they had used a self-test (e.g. testing for sexually transmitted diseases).

A weakness of this study may be that, because of its cross-sectional design, conclusions about causality cannot be drawn. Nonetheless, we believe the design is appropriate because the primary aim of this survey was to investigate the prevalence of self-testing and its correlates with socio-demographic variables.

## Conclusion

In conclusion, 8.5% of the 2.527 German participants of a representative face-to-face plus paper-pencil survey indicated having used a self-test to diagnose a disease or a disease risk in the past. Although the prevalence rates are lower in Germany than in the Netherlands and the UK, the need for and actual use of self-tests could increase in Germany in the future, given the growing number and diversity of available diagnostic self-tests [3], the ongoing rise of self-care and personal health practices, and the current shortage of physicians [24], especially in the rural areas of Germany [25]. As for several other self-care activities, the topic of self-testing also has a great practical and sociopolitical relevance, which is also reflected in current studies on the use of other direct-to-consumer and over the counter personal health products [26]. Thus, ethical and political considerations as well as new legal regulations are needed regarding the question whether certain self-tests should be promoted in the scope of prevention campaigns as it has been done in other European countries (e.g. by the Dutch Kidney Association or the Municipal Health). Next to self-tests for kidney diseases or chlamydia the pro’s and con’s regarding the promotion of self-tests for other diseases such as colorectal cancer should also be discussed, given the fact that more than 62.000 people in Germany were diagnosed with colorectal cancer in 2012 [27]. Furthermore, in future studies situational and health psychological predictors of self-testing should be investigated in more detail. Finally, the motives of conducting a self-test rather than consulting a health professional must still be investigated, as does the self-tester’s follow-up behavior after receiving the test results.

## Compliance with ethical standards

All procedures performed in studies involving human participants were in accordance with the ethical standards of the institutional and/or national research committee and with the 1964 Helsinki declaration and its later amendments or comparable ethical standards. The ethical approval for this study was given by the ethics committee of the Medical Faculty of the University of Leipzig in Germany (file number: 063-14-10032014).

## Supporting information

S1 Dataset Self-testing. (SAV)

S1 Text Questionnaire in English. (DOCX)

S2 Text Questionnaire in German. (DOCX)
