# Supplementary material for: Socio-demographic, health-related, and individual correlates of diagnostic self-testing by lay people: Results from a representative survey in Germany
Source: PLoS One. 2017 Nov 30;12(11):e0188653. doi: 10.1371/journal.pone.0188653 (PMC5708746; doi:10.1371/journal.pone.0188653)
Supplement: S2 Text — (DOCX) [file pone.0188653.s003.docx]

***Self-efficacy* was measured with the Short Scale for Measuring General Self-efficacy Beliefs (**[**ASKU, Beierlein, Kovaleva, Kemper, & Rammstedt, 2012**](#_ENREF_1)**).**

Die folgenden Aussagen können mehr oder weniger auf Sie zutreffen. Bitte geben Sie bei jeder Aussage an, inwieweit diese auf Sie persönlich zutrifft.

|  | trifft gar nicht zu | trifft wenig zu | trifft etwas zu | trifft ziemlich zu | trifft voll und ganz zu |
| --- | --- | --- | --- | --- | --- |
| 1. In schwierigen Situationen kann ich mich auf meine Fertigkeiten verlassen. | 🞏1 | 🞏2 | 🞏3 | 🞏4 | 🞏5 |
| 1. Die meisten Probleme kann ich aus eigener Kraft gut meistern. | 🞏1 | 🞏2 | 🞏3 | 🞏4 | 🞏5 |
| 1. Auch anstrengende und komplizierte Aufgaben kann ich in der Regel gut lösen. | 🞏1 | 🞏2 | 🞏3 | 🞏4 | 🞏5 |

***Physical and mental fatigue* was assessed with the personal burnout scale of the Copenhagen Burnout Inventory (**[**CBI, Kristensen, Borritz, Villadsen, & Christensen, 2005**](#_ENREF_3)**).**

***Kreuzen Sie bitte das Kästchen an, das Ihrer Zustimmung am besten entspricht.***

|  | nie/  fast nie | selten | manchmal | oft | immer |
| --- | --- | --- | --- | --- | --- |
| 01 Wie häufig fühlen Sie sich müde? | 🞏1 | 🞏2 | 🞏3 | 🞏4 | 🞏5 |
| 02 Wie häufig sind Sie körperlich erschöpft? | 🞏1 | 🞏2 | 🞏3 | 🞏4 | 🞏5 |
| 03 Wie häufig sind Sie emotional erschöpft? | 🞏1 | 🞏2 | 🞏3 | 🞏4 | 🞏5 |
| 04 Wie häufig denken Sie: “Ich kann nicht mehr?” | 🞏1 | 🞏2 | 🞏3 | 🞏4 | 🞏5 |
| 05 Wie häufig fühlen Sie sich ausgelaugt? | 🞏1 | 🞏2 | 🞏3 | 🞏4 | 🞏5 |
| 06 Wie häufig fühlen Sie sich schwach und krankheitsanfällig? | 🞏1 | 🞏2 | 🞏3 | 🞏4 | 🞏5 |

***Health satisfaction* was assessed within the module ‘General Life Satisfaction’ of the ‘Questions on Life Satisfaction Modules’ (**[**FLZM, Henrich & Herschbach, 2000**](#_ENREF_2)**).**


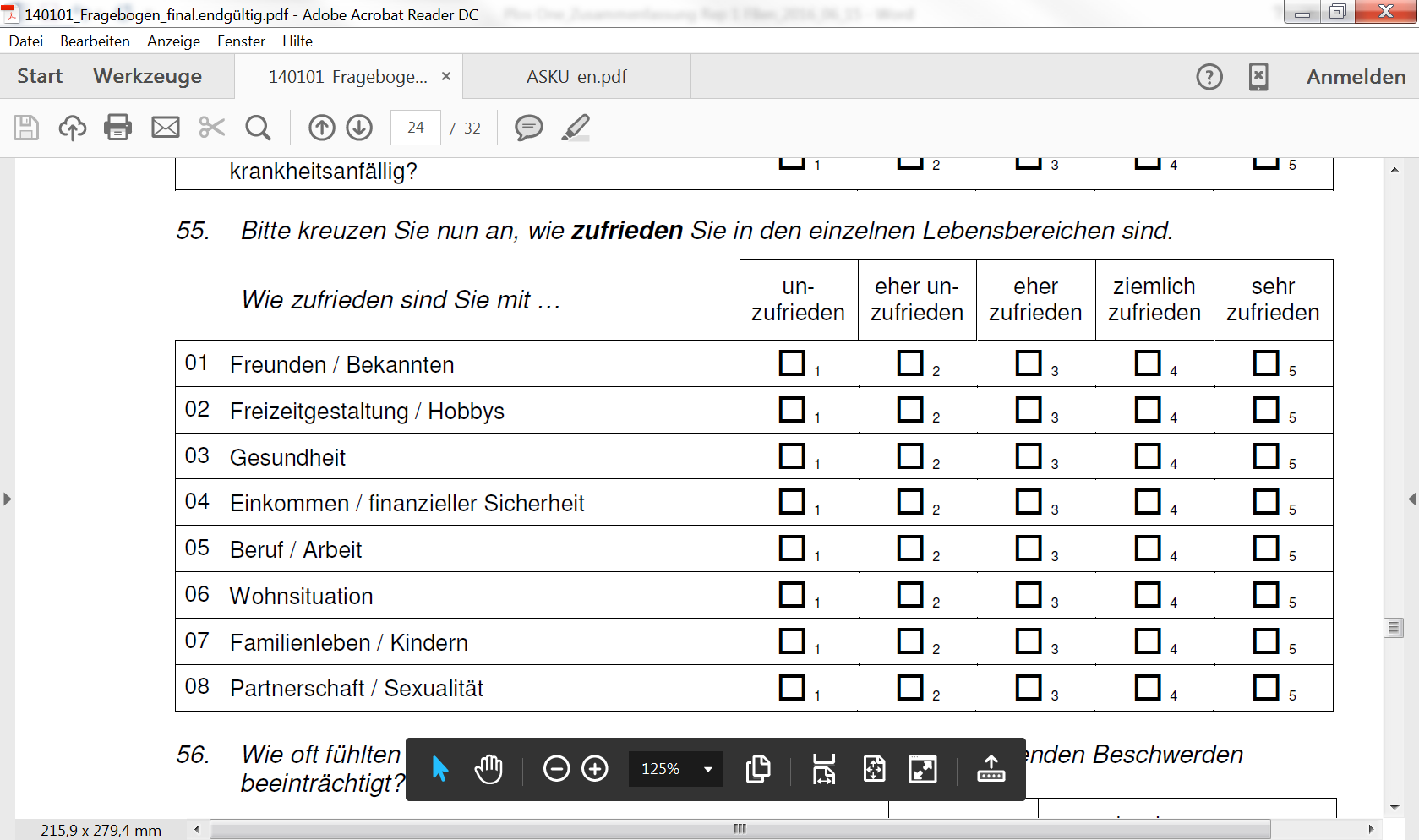


**Fragen zu Selbsttests, die zur Feststellung einer Erkrankung oder eines Erkrankungsrisikos angewendet werden können**

**Was ist ein „Selbsttest“?**

Bei einem „Selbsttest“ wird selbständig und ohne die Anwesenheit von medizinischem Personal eine Körperprobe entnommen (z.B. Blut, Urin, Stuhl oder Speichel). Diese Körperprobe wird auf eine Erkrankung oder ein Erkrankungsrisiko untersucht. Wichtig ist auch, dass ein „Selbsttest“ auf eigene Initiative – also nicht durch die Empfehlung eines Arztes – durchgeführt wird.

Ein Blutdruckmessgerät ist in diesem Sinne kein Selbsttest, da es keine Körperproben verwendet.

**1a. Haben Sie jemals einen Selbsttest gemacht/ einen Selbsttest machen lassen?**

- Nein, davon habe ich vor dieser Befragung noch nichts gewusst. *(Weiter mit Frage 2a.)*
- Nein, darüber habe ich noch gar nicht nachgedacht. *(Weiter mit Frage 2a.)*
- Nein, aber darüber habe ich schon einmal nachgedacht*(Weiter mit Frage 2a.)*
- Ja, nämlich **(Bitte ankreuzen, Mehrfachangaben möglich)**

1. Erbkrankheit(en)
2. Erkrankungen der Schilddrüse
3. Erkrankungen der Niere
4. Prostatakrebs
5. Darmkrebs
6. Diabetes
7. Allergien
8. Laktoseintoleranz
9. Glutenintoleranz
10. Cholesterol/Cholesterin
11. Blutarmut
12. Blutgerinnung
13. Influenza/Grippe
14. Pfeiffer(sches) Drüsenfieber
15. Hepatitis B oder C
16. Helicobacter pylori
17. Chlamydien
18. Syphilis
19. HIV-Infektion
20. Vaginalinfektion oder Hefepilzinfektion
21. Harnwegsinfektion
22. Fruchtbarkeit des Mannes
23. Fruchtbarkeit der Frau
24. Eisprung
25. Schwangerschaft
26. Verlust von Fruchtwasser
27. Menopause
28. Allgemeines Testkit, nämlich ……………………………………………… **(offene Angabe)**
29. Testkit für bestimmte Erkrankungen, nämlich …………………….…… **(offene Angabe)**
30. Andere Tests, nämlich ……………………………………………….. **(offene Angabe)**

**1b. *(Filter: Nur wenn 1a=Ja)***
**Wie und wo hatten Sie diese/n Selbsttest/s durchgeführt?
(Bitte machen Sie bei „…“ Ihre eigenen Angaben)**

| Welchen  Selbsttest  haben Sie  durchgeführt? | Wie oft haben Sie den Test bisher durchgeführt? | Haben Sie direkt danach das Ergebnis erhalten oder musste die Probe versendet werden? | Wo haben Sie den Test erworben? | Wo haben Sie den Test durchgeführt? |
| --- | --- | --- | --- | --- |
| **…** | … Mal | - Ergebnis gleich erhalten - Probe wurde versendet | **…** | **…** |
| **…** | … Mal | - Ergebnis gleich erhalten - Probe wurde versendet | **…** | **…** |
| **…** | … Mal | - Ergebnis gleich erhalten - Probe wurde versendet | **…** | **…** |
| **…** | … Mal | - Ergebnis gleich erhalten - Probe wurde versendet | **…** | **…** |
| **…** | … Mal | - Ergebnis gleich erhalten - Probe wurde versendet | **…** | **…** |

**2a. Beabsichtigen Sie Selbsttests in Zukunft anzuwenden?**

- Definitiv nicht. (*Abschluss der Befragung)*
- Wahrscheinlich nicht. (*Abschluss der Befragung*)
- Vielleicht.
- Wahrscheinlich.
- Definitiv.

**2b. Bitte geben Sie alle Selbsttests an, die Sie in Betracht ziehen würden:**

…………………………..…………………………..…………………………..……………………….…………………………..…………………………..…………………………..……………………….

…………………………..…………………………..…………………………..……………………….

**References**

Beierlein, C., Kovaleva, A., Kemper, C. J., & Rammstedt, B. (2012). *Ein Messinstrument zur Erfassung subjektiver Kompetenzerwartungen: Allgemeine Selbstwirksamkeit Kurzskala (ASKU) [Short Scale for Measuring General Self-efficacy Beliefs (ASKU)]*. Mannheim, Germany.

Henrich, G., & Herschbach, P. (2000). Questions on Life Satisfaction (FLZM) - A short questionnaire for assessing subjective quality of life. *European Journal of Psychological Assessment, 16*(3), 150-159.

Kristensen, T. S., Borritz, M., Villadsen, E., & Christensen, K. B. (2005). The Copenhagen Burnout Inventory: A new tool for the assessment of burnout. *Work & Stress, 19*(3), 192-207. doi: 10.1080/02678370500297720
